# From the Cochrane Library: Topical Tacrolimus for Atopic Dermatitis

**DOI:** 10.2196/31838

**Published:** 2021-10-28

**Authors:** Austin Hamp, Jarett D Anderson, Torunn E Sivesind, Mindy D Szeto, Jade Cury-Martins

**Affiliations:** 1 Arizona College of Osteopathic Medicine Midwestern University Glendale, AZ United States; 2 Department of Dermatology University of Colorado Anschutz Medical Campus Aurora, CO United States; 3 Department of Dermatology University of São Paulo São Paulo Brazil

**Keywords:** atopic dermatitis, eczema, calcineurin inhibitor, tacrolimus, lymphoma, dermatology, dermatitis

Among chronic skin disorders, atopic dermatitis demonstrates the greatest negative impact on quality of life for both affected patients and their families, and is associated with anxiety, guilt, and depression [1]. Emotional stress from uncontrolled eczema can be displaced on coworkers, students, and teachers, affecting the entire community. Thus, effective eczema management potentially has far-reaching benefits.

Eczema is commonly treated with topical corticosteroids (TCS), the long-term use of which may cause dermal atrophy, striae, and hypertrichosis, among other adverse effects [2]. Additionally, patients may experience treatment-resistant eczema. Therefore, cost-effective alternative treatments with fewer side effects may be prudent. A 2015 Cochrane review, titled “Topical tacrolimus for atopic dermatitis,” assessed the efficacy and safety of the topical calcineurin inhibitors (TCIs) tacrolimus and pimecrolimus in comparison to conventional treatments (20 randomized controlled trials, n=5885) [2]. Significant drug comparison findings are summarized in Table 1. This review found convincing evidence that tacrolimus 0.1% was significantly more effective than low-potency TCS, pimecrolimus 1%, and tacrolimus 0.03% at improving physician- and patient-assessed appearance of eczema-affected skin in many settings. While results were mainly obtained using the subjective measures mentioned previously, several trials included in the review indicated that tacrolimus significantly improved objective measures such as the Eczema Area and Severity Index, quality of life, and Scoring Atopic Dermatitis (SCORAD) when compared to certain TCS in various settings. However, lack of data on these secondary outcomes limited the completeness of evidence. Unfortunately, since the publication of this Cochrane review, only 1 randomized controlled trial analyzing the efficacy of TCIs has been published [3]. Despite this study validating the findings of this Cochrane review, further research is warranted to investigate the true relationship between TCS and TCIs utilizing objective standardized criteria.

One notable side effect found in this review was burning and pruritus experienced in the first days of TCI treatment, which was most pronounced when comparing topical tacrolimus with TCS (risk ratio 2.48, 95% CI 1.96-3.14, 5 studies, n=1883, high-quality evidence). These symptoms were mild and transient, and generally did not lead to discontinuation of treatment. There were no reported TCI-related cases of skin atrophy nor evidence of increased risk of malignancies.

Despite the minimal side-effects profile, a black box warning for topical tacrolimus remains due to concern for malignancies associated with systemic absorption, which is low when administered topically [4]. Nevertheless, patients with rare skin diseases such as Netherton syndrome and lamellar ichthyosis should be cautioned, as systemic absorption of TCIs was noted in these patients [2]. Reassuringly, a recent 10-year prospective longitudinal study following 7954 children with atopic dermatitis who used topical tacrolimus for ≥6 weeks reported no cases of lymphoma [5].

The efficacy of tacrolimus was further substantiated with a recent 2020 study reporting that tacrolimus 0.1% was more likely to achieve clear or almost clear skin at 28 to 42 days versus vehicle (hazard ratio 1.74, 95% CI 1.13-3.05) based on the Investigator’s Static Global Assessment [6]. Eczema results in psychological stress to the patient, as well as to the patient’s family and community. Options with a favorable side-effects profile for treatment-resistant eczema or for patients who are intolerant to TCS are desirable [1]. Tacrolimus is a suitable and effective alternative under these conditions. Thus, tacrolimus appears to be an effective treatment for eczema.

**Table 1:** Eczema drug efficacy comparisons with respective measurements, results, risk ratios (RRs), and quality of evidence.

Comparison	Measurement^a^	Result	RR	Quality of evidence^b^
Tacrolimus 0.1% vs low-potency TCS^c^ (follow-up: mean 3 weeks)	Physician’s assessment	Tacrolimus 0.1% superior	RR 3.09, 95% CI 2.14-4.45, 1 RCT^d^, n=371	Moderate
Tacrolimus 0.1% vs moderate-potency TCS (follow-up: 6 months)	Physician’s assessment	Tacrolimus 0.1% marginally better	RR 1.32, 95% CI 1.17-1.49, 2 RCTs, n=506	Moderate
Tacrolimus 0.1% vs pimecrolimus 1% (follow-up: mean 6 weeks)	Physician’s assessment	Tacrolimus 0.1% superior	RR 1.80, 95% CI 1.34-2.42, 2 RCTs, n=506	Moderate
Tacrolimus 0.03% vs tacrolimus 0.1% (follow-up: 3-12 weeks)	Physician’s assessment	Tacrolimus 0.1% superior	RR 0.82, 95% CI 0.72-0.92, 6 RCTs, n=1640	High
Tacrolimus 0.1% vs moderate to high potency TCS (follow-up: mean 3 weeks)	Physician’s assessment	No difference	RR 0.95, 95% CI 0.78-1.16, 1 RCT, n=377	Low
Tacrolimus 0.1% vs moderate to high potency TCS (follow-up: mean 6 months)	Participant’s self-assessment	Marginal benefit favoring tacrolimus 0.1%	RR 1.21, 95% CI 1.13-1.29, 1 RCT, n=974	Low
Tacrolimus 0.03% vs mild-potency TCS (follow-up: mean 3 weeks)	Physician’s assessment	Tacrolimus 0.03% superior	RR 2.58, 95% CI 1.96-3.38, 2 RCTs, n=790	Moderate

^a^Physicians and participants rate skin improvement in a subjective manner*.* Though subjective, these tools are commonly used to assess treatment efficacy. For example, skin improvement is evaluated as excellent improvement (>90% improvement), marked improvement (75%-89%), or moderate improvement (50%-74%) from the participant’s or physician’s viewpoint [4].

^b^High quality: further research is very unlikely to change the confidence in the estimate of effect; moderate quality: further research is likely to have an important impact on the confidence in the estimate of effect and may change the estimate; low quality: further research is very likely to have an important impact on the confidence in the estimate of effect and is likely to change the estimate; very low quality: very uncertain about the estimate.

^c^TCS: topical corticosteroids.

^d^RCT: randomized controlled trial.

## Abbreviations

SCORAD: Scoring Atopic Dermatitis
TCI: topical calcineurin inhibitor
TCS: topical corticosteroids

## References

[1] Na Chan Ho, Chung Janice, Simpson Eric L (2019). Quality of Life and Disease Impact of Atopic Dermatitis and Psoriasis on Children and Their Families. Children (Basel).

[2] Cury Martins Jade, Martins Ciro, Aoki Valeria, Gois Aecio F T, Ishii Henrique A, da Silva Edina M K (2015). Topical tacrolimus for atopic dermatitis. Cochrane Database Syst Rev.

[3] Perälä Miia, Ahola Maria, Mikkola Tytti, Pelkonen Anna S, Remitz Anita, Mäkelä Mika J (2020). Young children with moderate-to-severe atopic dermatitis can be treated safely and effectively with either topical tacrolimus or mild corticosteroids. Acta Paediatr.

[4] Lam Megan, Zhu Jie Wei, Tadrous Mina, Drucker Aaron M (2021). Association Between Topical Calcineurin Inhibitor Use and Risk of Cancer, Including Lymphoma, Keratinocyte Carcinoma, and Melanoma: A Systematic Review and Meta-analysis. JAMA Dermatol.

[5] Paller Amy S, Fölster-Holst Regina, Chen Suephy C, Diepgen Thomas L, Elmets Craig, Margolis David J, Pollock Brad H (2020). No evidence of increased cancer incidence in children using topical tacrolimus for atopic dermatitis. J Am Acad Dermatol.

[6] Fahrbach Kyle, Tarpey Jialu, Washington Evelien Bergrath, Hughes Rachel, Thom Howard, Neary Maureen P, Cha Amy, Gerber Robert, Cappelleri Joseph C (2020). Crisaborole Ointment, 2%, for Treatment of Patients with Mild-to-Moderate Atopic Dermatitis: Systematic Literature Review and Network Meta-Analysis. Dermatol Ther (Heidelb).

